# An Experimental Design Approach for Producing Curcumin-Loaded Solid Lipid Nanoparticles

**DOI:** 10.3390/ph18040470

**Published:** 2025-03-27

**Authors:** Ongun Mehmet Saka, Cemre İrem Aygüler, Neval Sevinç Özdemir, Bilge Sürücü, Egemen Çakırlı, Emirhan Nemutlu, Gülen Melike Demirbolat

**Affiliations:** 1 Department of Pharmaceutical Technology, Faculty of Pharmacy, Ankara University, 06560 Ankara, Türkiye; omsaka@gmail.com; 2 Department of Pharmaceutical Technology, Faculty of Pharmacy, Acıbadem Mehmet Ali Aydınlar University, 34752 Istanbul, Türkiye; cemre.ayguler@acibadem.edu.tr; 3 Department of Pharmaceutical Basic Sciences, Faculty of Pharmacy, Acıbadem Mehmet Ali Aydınlar University, 34752 Istanbul, Türkiye; neval.sevinc@acibadem.edu.tr; 4 ACU Biomaterials Center, Acıbadem Mehmet Ali Aydınlar University, 34752 Istanbul, Türkiye; 5 Department of Pharmaceutical Technology, Faculty of Pharmacy, İstanbul University, 34116 Istanbul, Türkiye; ecz.bilgesurucu@gmail.com; 6 Department of Pharmaceutical Biotechnology, Faculty of Pharmacy, Acıbadem Mehmet Ali Aydınlar University, 34752 Istanbul, Türkiye; egemen.cakirli@acibadem.edu.tr; 7 Department of Genetics and Bioengineering, Faculty of Engineering, Yeditepe University, Atasehir, 34755 Istanbul, Türkiye; 8 Department of Analytical Chemistry, Faculty of Pharmacy, Hacettepe University, 06800 Ankara, Türkiye; enemutlu@hacettepe.edu.tr

**Keywords:** design of experiment, curcumin, solid lipid nanoparticles, nanoparticles

## Abstract

**Background/Objectives**: Curcumin has well-established efficacy in a variety of disorders due to its prominent antioxidant, antiaging, anti-inflammatory, chemosensitizing, and anticancer activities. Despite its numerous benefits, curcumin exhibits low bioavailability mainly due to its poor solubility, poor absorption, rapid metabolism, and quick excretion, consequently limiting its clinical applications. In this study, we investigated the most convenient ingredients in SLNs to enhance curcumin’s solubility by examining the effects of multiple independent variables simultaneously using an experimental design. **Methods**: After curcumin’s saturation solubility was investigated, SLN formulations were produced. The optimum formulation was determined with the help of experimental design. The SLNs were characterized in terms of the particle size and distribution, zeta potential, shape, entrapment efficiency, drug loading capacity, and drug release. The cell viability and cell internalization were also evaluated. **Results**: An impressive synergistic effect was achieved with the combination of Brij and Gelucire 48/16, which increased curcumin’s solubility in water by 452.5 times. Curcumin-loaded SLNs were successfully produced with a spherical shape and particle size of 389.3 ± 9.95 nm. The encapsulation efficiency was directly proportionate to the amount of curcumin and the stirring speed. Curcumin in the SLNs entered the cancer cells more easily than curcumin alone. **Conclusions**: Our results demonstrate that the quantity of surfactant is a significant factor influencing the efficiency of drug loading. Finally, the 3:1 (Brij–Gelucire48/16) ratio markedly enhanced the loading efficiency. The cellular internalization and, consequently, the anticancer efficacy against adenocarcinomic human alveolar basal epithelial cells were improved with SLNs. This could be a promising approach for lipid-based colloidal drug delivery systems.

## 1. Introduction

Curcumin is used in a wide range of pharmaceutical applications, including antioxidant, anti-inflammatory, antimicrobial, and anticancer drugs. In particular, the use of curcumin for cancer treatment inhibits carcinogenesis, angiogenesis, and tumor growth, while it reduces chemotherapy and radiotherapy side effects [1]. It is a versatile drug that can be used for treating many conditions such as cough/inflammation, respiratory diseases, flu, sinusitis, liver disorders, rheumatism, abdominal pain, burn wounds, neurological disorders, and tissue regeneration [2,3]. In addition to its potential usage, its effect on H. pylori treatment was also demonstrated recently [4]. However, this chemical generally shows limited serum levels after oral administration, due to its high susceptibility to metabolic degradation prior to absorption within the intestine and liver. Moreover, it is insoluble in water and unstable under light and oxygen. Because it is a light-sensitive substance, it can undergo photodegradation [5,6]. In the process of finding a solution to optimize curcumin administration, the use of nanotechnology has become a very promising and significant approach, especially since conventional methods for enhancing the water solubility of drugs typically lead to poor pharmacokinetics and low bioavailability [7].

With the help of nanotechnology, drug carriers serve as drug depot systems that enable the delivery of poorly water-soluble drugs, bypassing the liver, preventing first-pass metabolism, and, thus, increasing bioavailability while minimizing side effects. To overcome the aforementioned limitations related to curcumin, several attempts focusing on nanotechnology have been made such as polymeric micelles, nanoparticles, liposomes, cyclodextrins, biodegradable hydrogels, and microemulsions [8]. Solid lipid nanoparticles (SLNs), a type of colloidal carrier based on nanotechnology, consist of a solid lipid content (trimyristin, tristearin, trilaurin, stearic acid, glyceryl caprate, theobroma oil, triglyceride coconut oil, 1-octadecanol, glycerol behenate, glycerol palmitostearate, cetylpalmitate wax, etc.); a surfactant (tween, polysorbates, lecithins, etc.); and an incorporated drug. The SLN structures are stabilized by suitable surfactant(s) [9] and offer distinct advantages such as biocompatibility, biodegradability, nontoxicity, high drug payload capacity, a longer shelf life, ease of scalability, modifiable release patterns, and coalescence resistance. Furthermore, they are efficient carriers that improve solubility, increase bioavailability, and bypass first-pass metabolism [10]. Researchers have made significant efforts to enhance the bioavailability of curcumin by developing various novel drug delivery systems, including solid lipid nanoparticles. However, curcumin has not yet been licensed as a drug. Hassanzadeh et al. reviewed the obstacles against the commercialization of curcumin as a drug [11]. Bypassing these abovementioned limitations of curcumin still requires more applicative research despite a significant number of curcumin-based formulations.

Considering all this information, we proposed that combining traditional solubility enhancement methods with nanotechnology could be an effective solution to the challenges associated with curcumin. Therefore, the aim of the present study was to systematically evaluate the effect of cosolvency, complexation, and micellar solubilization approaches on curcumin solubility as well as to incorporate this approach into nanotechnology-based drug carrier systems, like SLNs. After the selection of the most convenient excipients, curcumin-loaded SLNs were produced and characterized in accordance with formulation optimization studies.

## 2. Results

### 2.1. Saturation Solubility Study

**Figure 1:**
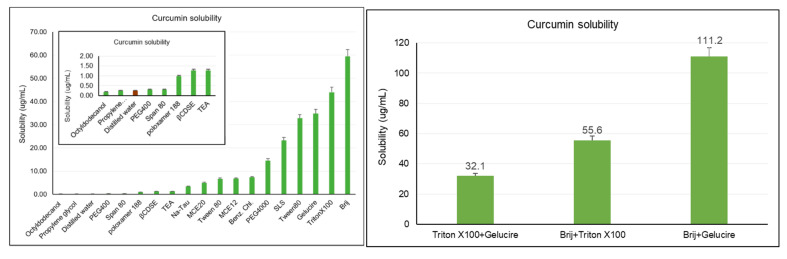
Solubility values of curcumin in different solutions.

Curcumin is practically insoluble in water as its water solubility is only 0.6 µg/mL [12]. Various methods can be used to enhance the water solubility of poorly soluble drugs, including cosolvency, complexation, micellar solubilization, micronization, nanosuspension, SLNs, and solid dispersion [13]. In this study, we employed cosolvency, complexation, and micellar solubilization to investigate the most convenient excipients for curcumin-loaded nanocarrier formulation. The solubility of curcumin in different compounds was evaluated in order to increase the drug solubility and payload in the formulation. The tendency of compounds to solubilize the drug determined our choice of chemicals (either surfactants or cosolvents). To evaluate the effect of micellar solubilization, Brij 35 (polyoxyethylene (23) lauryl ether), TritonX100 (polyoxyethylene octyl phenyl ether), tween 80 (polyethylene glycol sorbitan monooleate), span 80 (sorbitane monooleate), triethanol amine (TEA), Gelucire 48/16 (polyoxyl-32 stearate), poloxamer 188 (poly(ethylene glycol)-block-poly(propylene glycol)-block-poly(ethylene glycol)), sodium lauryl sulfate (SLS; anionic surfactant), and benzalkonium chloride (Benz. Ch.; cationic surfactant) were used as surfactants. Propylene glycol (PG), polyethylene glycol 400, polyethylene glycol 4000, octyldodecanol (OD), macrogol cetostrearyl ether 12 (MCE12), and macrogol setotrearil ether 20 (MCE20) were used as cosolvents. β cyclodextrine sulfobutyl ethers (βCDSE) were used to evaluate the effect of complexation on curcumin’s solubility. Sodium taurocholate (Na-Tau) acted as a detergent. Therefore, apart from distilled water, 18 different compounds were evaluated with respect to how effective curcumin’s solubility was, as shown in Figure 1. It could be seen that most of these compounds could not improve solubility to a value greater than 10 µg/mL. The top six surfactants that enhanced curcumin’s solubility in water the most were Brij, TritonX100, Gelucire, Tween80, SLS, and PEG4000, in order from most to least effective.

### 2.2. Preliminary Study

Preliminary experimental runs were conducted to reduce the critical process parameters prior to starting the DoE research. For this purpose, ten different formulations were evaluated in terms of the lipid amount, drug amount, and surfactant ratio. The particle sizes of the samples ranged from 1039.8 ± 208.22 nm (micrometer scale) to 307.70 ± 89.10 nm. A PDI of 0.3 or below indicates a homogeneous population of lipid carriers [14]. The PDI of the formulations were less than 0.3, with the exception of two, and the ZP value in the SLN dispersion represents the strength of electrostatic attractions between adjacent, similarly charged particles. The particles of the tested suspension must experience repulsion in order to prevent particle aggregation and, thus, guarantee the absence of flocculation [15]. The drug delivery literature frequently defines dispersions with ZP values of >±30 mV as extremely stable [16]. All formulations were found to be extremely stable, with ZP values less than −30 mV.

Among all of the experimental responses, the particle size was deemed to be the most sensitive one. Increasing the lipid amount and/or the drug amount in the formulation resulted in the generation of bigger particles. Small particle sizes (>400 nm) were only obtained using 100 mg of stearic acid, which was kept the same for the subsequent DoE study. During the purification step, the precipitation of 10 mg curcumin could be observed by the naked eye in the formulations after centrifugation due to its low water solubility. It was very challenging to completely eliminate this from the formulations. Therefore, this 10 mg of curcumin was eliminated during the subsequent experiments.

### 2.3. Experimental Design

An orthogonal CCD across 17 runs showed the critical factors (curcumin amount, surfactant ratio, and stirring speed) and their corresponding responses on the PS, ZP, EE%, and DL% (Table 1). The coefficient of each variable, including the quadratic terms and interactions of the responses, is summarized in Table 2. When the values are positive, the value of the parameter under examination increases in conjunction with the independent variables. Conversely, when the value is negative, an inverse relationship between the variables in the equation exists [17]. As a result of the ANOVA, the established statistical models highlighted the significance of the main effects, quadratic terms, and interactions among factors. The stirring speed (b3) significantly affects the PS and DL%, while interactions like the curcumin amount and stirring speed (b1 × b3) influence the EE%.

**Figure 2:**
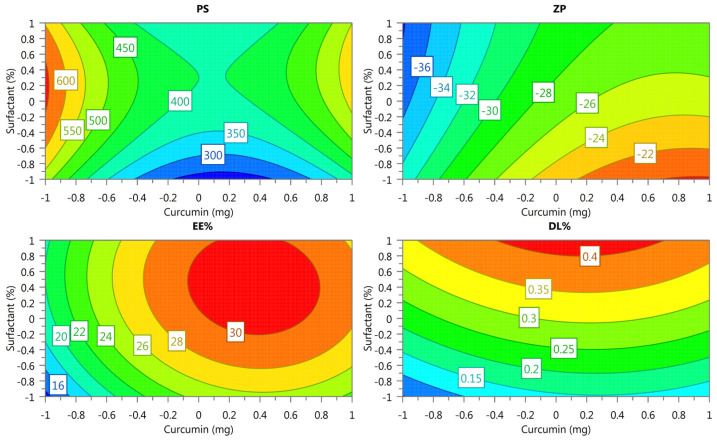
The response surface graph DL%, PS, ZP, and EE% as functions of the X_1_ and X_2_ independent variables. The values were obtained by setting the stirring time to 1000 rpm.

This study highlights that the surfactant ratio plays a crucial role in improving the EE% and DL%, while the stirring speed and curcumin amount have a stronger impact on the PS. Therefore, response surface plots for the DL%, PS, ZP, and EE% were created as functions of the X_1_ and X_2_ independent variables (Figure 2). The surface plot analysis reveals significant insights into the effects of the curcumin-to-surfactant ratio on the response variables. For the PS, the smallest sizes were achieved at lower levels of surfactant, with curcumin having a comparatively moderate impact. The ZP becomes more negative as the surfactant ratio increases, indicating improved colloidal stability, while higher levels of curcumin lead to more positive ZP values. The EE% improves significantly with higher surfactant levels, especially when curcumin is kept at moderate levels. Lastly, the DL% is maximized at higher surfactant levels, irrespective of the curcumin level. These findings emphasize the dominant role of the surfactant ratio in enhancing the EE% and DL%, while the PS is influenced more strongly by curcumin and stirring speed interactions. These insights provide a clear direction for optimizing critical factors to achieve desired experimental outcomes. Therefore, optimization efforts were focused on balancing these parameters to achieve a smaller PS and ZP and higher EE% and DL% (Table 3).

**Figure 3:**
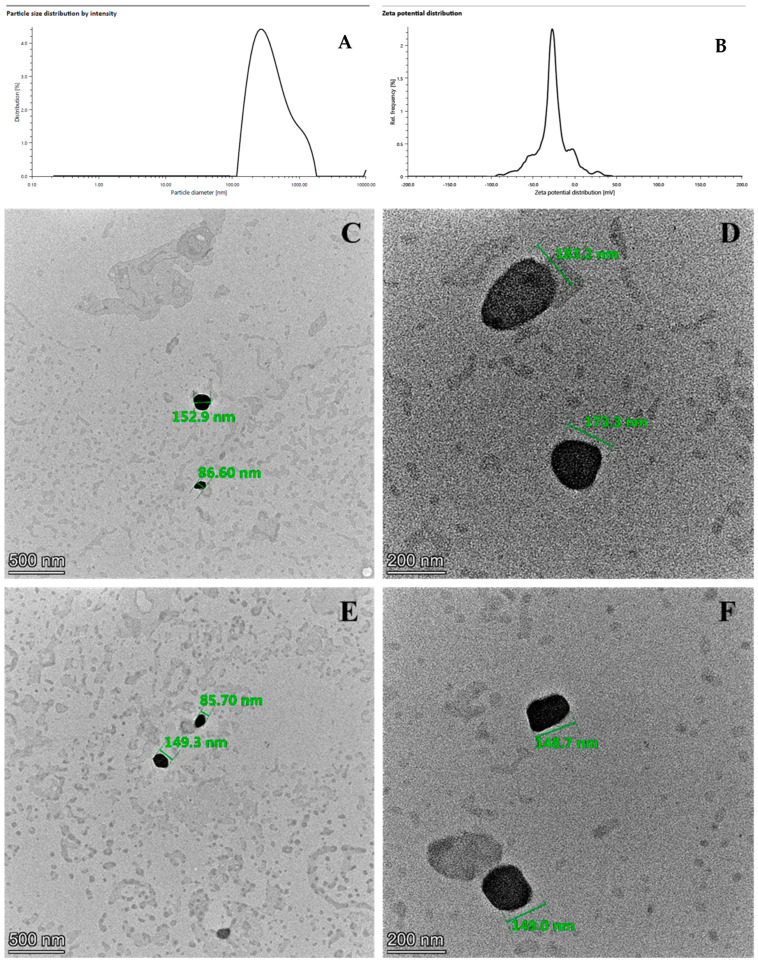
The particle size and distribution graph: (**A**) Zeta potential diagram; (**B**) TEM images of the optimum formulation; (**C**,**D**) blank SLNs; (**E**,**F**) curcumin-loaded SLNs. Particle sizes from top to bottom: 152.9 nm, 86.60 nm (**C**); 183.2 nm, 173.3 nm (**D**); 85.70 nm, 149.3 nm (**E**); 148.7 nm, 149.0 nm (**F**).

The optimum formulation was produced using 300 mg of Brij, 100 mg of Gelucire 48/16 in 10 mL of distilled water as the aqueous phase, and 100 mg of stearic acid dissolved in 1 mL of ethanol after ultrasonication for a while. A specific amount of curcumin (4.375 mg) was dissolved in 1 mL of ethylacetate. The organic phase (2 mL in total) was added dropwise to the aqueous phase (45 °C) under magnetic stirring at 1000 rpm in 10 min. After the addition of the organic phase, the temperature was increased up to 75 °C and stirred overnight to evaporate the organic solvent. After the purification process (centrifugation at 60,000 rpm for 30 min), the optimum formulation was characterized. Both theoretical and experimental results are presented in Table 3. As a consequence of the preliminary study, the optimum formulation was already known to be within certain boundaries. The particle size and distribution and zeta potential graphs are illustrated in Figure 3A,B, respectively. This was confirmed by the experimental design, which showed that the calculated and measured values for the PS, ZP, and EE were identical. However, there was a discrepancy in the DL efficiency.

### 2.4. Morphological Analysis

Figure 3 displays TEM images of the dispersions of optimized drug-free (C&D) and drug-loaded (E&F) SLNs, confirming the smooth shape and submicron size of the particles. In the TEM images, particles of 86.6 nm to 183.2 nm in size were identified for the drug-free SLNs, while the size of the curcumin-loaded SLNs changed from 149.0 nm to 85.7 nm. During the sample preparation for the TEM images, the samples were completely dried. Because of the absence of solvent in the system, the particle sizes shown in the TEM images are smaller than those shown in DLS images.

### 2.5. In Vitro Release Study

**Figure 4:**
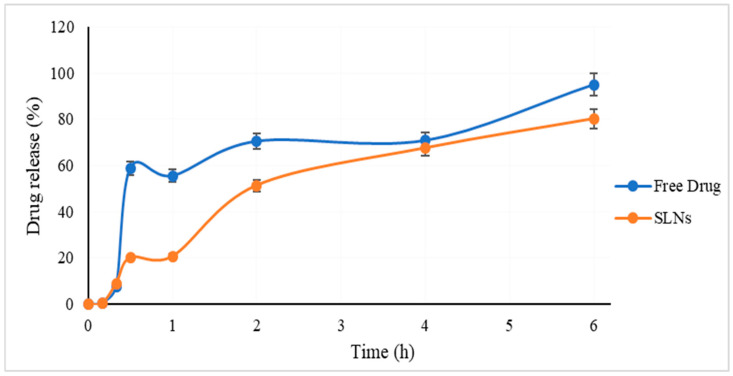
Release profile of free curcumin (blue line) and curcumin-loaded SLNs (orange line).

The drug release study was performed in both a PBS (pH 7.4)–ethanol mixture (80:20) and in PBS pH 7.4 with 0.1% *w*/*v* Tween80. When the latter medium was used, we obtained samples with yellowish colors, including curcumin solution in PBS with 0.1% *w*/*v* Tween80, even after 24 h, which was an indicator of incomplete release. Thus, the PBS–ethanol mixture was used as the medium. Figure 4 illustrates the cumulative release of curcumin as a percentage plotted as a function of time. It can be seen that the amount of curcumin in the curcumin solution reached approximately 60% during the first 30 min and 100% at the end of the experiment. In contrast, we also observed curcumin release from the SLNs in a sustained manner without any burst effects, reaching 80.1% after six hours. A drug release rate of 60% was achieved in the first 30 min for the curcumin ethanolic solution whereas the curcumin release from the SLNs reached 60% within 4 h. To elucidate the release behavior, kinetic models were applied.

When the release data were analyzed in terms of the release kinetics, curcumin release from the produced SLNs was fitted to the Higuchi release kinetics model since the related correlation coefficients were better than the others (seen in Table 4). Higuchi’s model characterizes drug release from an insoluble matrix as a time-dependent process [18]. Therefore, it was concluded that curcumin homogenously dispersed into the solid lipid matrix. There being no burst effect from the SLNs also pointed to the absence of curcumin accumulation on the outer surface of the SLNs, where the hydrophilic parts of the surfactants were located. It can be interpreted that the release mechanism relied on diffusion-controlled release.

### 2.6. Short-Term Stability

**Figure 5:**
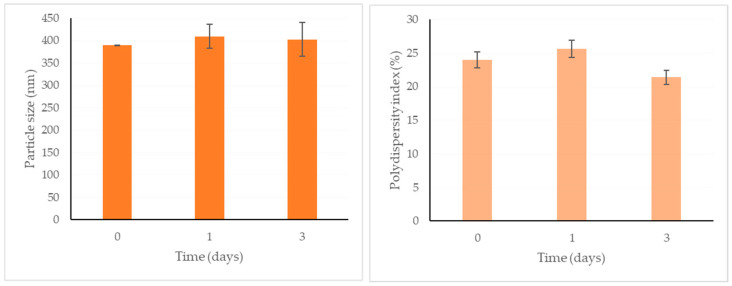
Changes in particle size and polydispersity index of curcumin-loaded SLNs within 3 days (*p* > 0.05: no significant difference).

The physical stability of the curcumin-loaded SLNs was also evaluated for 3 days (Figure 5). At the storage temperature, which was +4 °C ± 1, the particle size and polydispersity index was not statistically changed for the short time period.

### 2.7. Cell Viability

**Figure 6:**
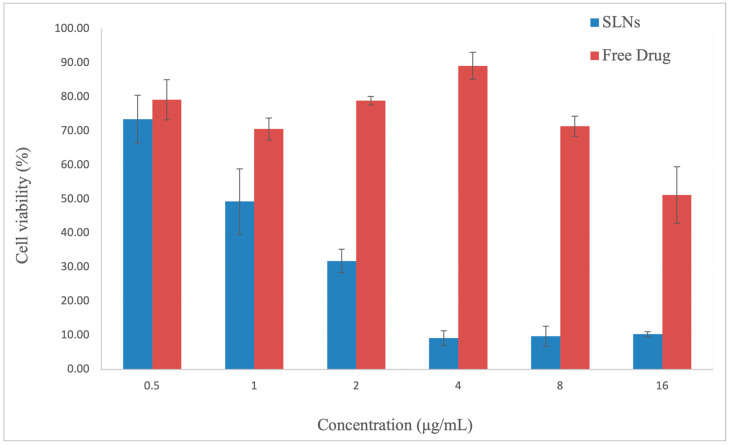
A549 cell viability (%) in response to treatment with free curcumin and curcumin-loaded SLNs for 24 h.

The effect of the free curcumin and curcumin-loaded SLNs on the A549 cell viability was evaluated using the MTT assay after 24 h of treatment. A pronounced decline in the cell viability was observed with increasing concentrations of SLNs. The highest cell viability percentages for each tested concentration were recorded when curcumin was applied in its free form (Figure 6). These findings suggest that curcumin exerts a greater effect on A549 adenocarcinoma cells when delivered via SLNs compared to in its free form.

### 2.8. Cellular Internalization

**Figure 7:**
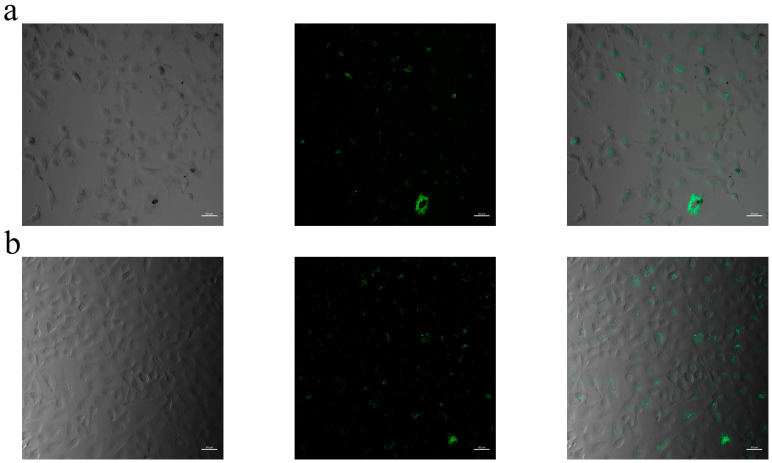

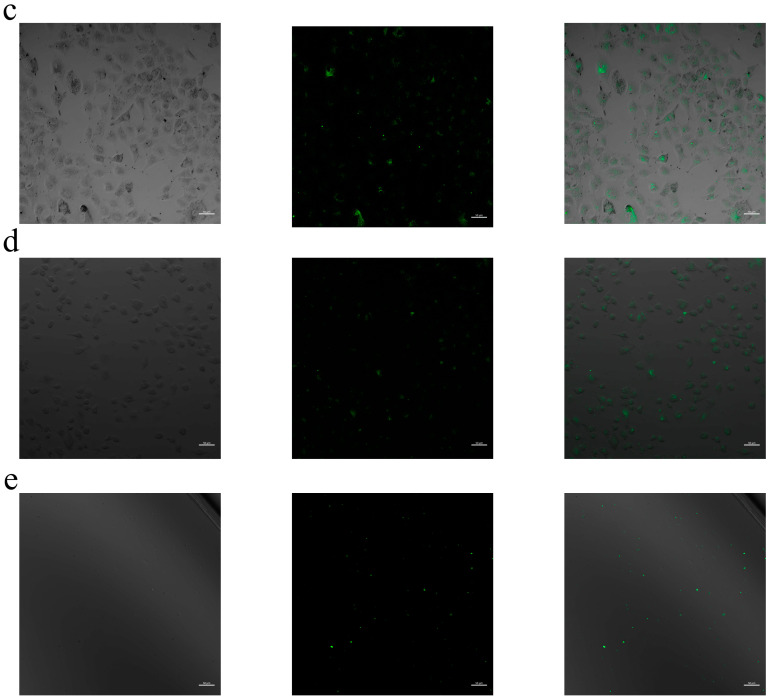
Fluorescent images of A549 cells treated with curcumin-loaded solid lipid nanoparticles (SLNs). Multi-panel images display brightfield, Alexa Fluor 488, and merged fluorescence images of A549 cells treated with different concentrations of curcumin SLNs: (**a**) 1.56 µg/mL, (**b**) 3.13 µg/mL, (**c**) 6.25 µg/mL, (**d**) 12.50 µg/mL, and (**e**) 25 µg/mL. The images illustrate the concentration-dependent uptake of curcumin SLNs by A549 cells (scale bar: 50 µm).

The internalization of SLN–curcumin into A549 cells was verified through cell internalization assays, demonstrating its effective uptake. Clear fluorescence intensity was seen at all concentrations when the cells were incubated with the curcumin-loaded SLNs (Figure 7). The cellular uptakes of the SLNs were highly concentration-dependent. Confocal microscopy imaging further revealed that SLN–curcumin was confined to the cytoplasm, without evidence of nuclear penetration. This result was consistent with the literature [19]. Moreover, the cells lost their regular morphology when SLN formulations containing curcumin concentrations of 6.25 µg/mL or higher were used. At higher concentrations, the cells became spherical and lost their viability, which was also in line with the cytotoxicity results.

## 3. Discussion

Poor solubility is a major barrier against good bioavailability, as is known. Although curcumin is a versatile drug and is used for various treatments, it suffers from poor solubility. The solubility of poorly soluble drugs can be improved using a variety of approaches, such as nanosuspensions, the use of surfactants or cosolvents, etc. In this study, we investigated the effects of different solubility enhancement approaches like surfactants and cosolvents with many different properties on the solubility of curcumin. The saturation solubility study of curcumin showed that the effect of cosolvency (using PG and PEG400) was observed at the lowest rate (more or less than its solubility in distilled water), and complexation (with βCDSE) or using bile salts (Na-Tau) only led to a moderate effect while that of micellar solubilization was the most prominent. The substances with low hydrophilic–lipophilic balance (HLB) values (octyldodecanol and Span80) did not have as much of an effect on the solubility of curcumin as those with a high HLB. The presence of PEG chains in the substance substantially increased the solubility of curcumin in water. For instance, tween 80 (a derivative of Span 80), containing a polyoxyethylene chain, increased the water solubility of curcumin by 100 times. Moreover, it was observed that the four substances with the highest effect on curcumin’s solubility contained a medium-chain PEG. On the other hand, the fact that a similar effect could not be obtained with PEG4000 may be related to its high PEG chain length. Our findings showed that Gelucire 48/16, Triton-X 100, and Brij 35 enhanced curcumin’s solubility by 142, 179, and 242 times, respectively.

Consequently, these surfactants can be considered excellent candidates for SLN formulation, as only minimal quantities are required in order to load the necessary amount of curcumin. We then evaluated combinations of two of these three surfactants, and the most impressive synergistic effect was achieved with Brij 35 and Gelucire 48/16, which increased curcumin’s solubility in water by 452.5 times (111.2 µg/mL), compared to that in distilled water (0.25 µg/mL). Similarly, Das et al., in their study on the solubility capacities of different Brij types (Brij 30, Brij 35, Brij 52, Brij 58, Brij 92, and Brij 97), reported that Brij-35 provided the maximum solubilization due to its high hydrophilicity [20]. The solubility of curcumin in Gelucire^®^ 48/16 solution, investigated by Shinde, showed similar results to those of our study [21]. On the other hand, the solubility of curcumin was found to be superior in a mixed surfactant solution in our study as compared to the solubility of an individual surfactant.

This highlighted the importance of using surfactants when preparing the formulation. Moreover, the binary system was found to be more beneficial. Using mixed surfactants was more efficient for improving the solubility of curcumin in this study. Surfactants can be used in many different nanocarrier systems. Li et al. produced oil-free self-assembled micelles with a smaller size and high entrapment efficiency using kolliphor RH 40 and Tween80 [22]. Compared to micelles, solid lipid nanoparticles show a high drug payload, and they might represent an optimal nanocarrier system due to their biocompatibility and biodegradability. The selection of the types and amount of ingredients in SLNs is raised in terms of them being critical quality attributes. Therefore, we used design of experiments to examine the effects of multiple independent variables simultaneously, thereby determining the optimum formulation conditions in order to ensure the desired product quality.

Encapsulating curcumin within its lipid phase can improve its bioaccessibility and chemical stability by physically isolating curcumin from reactants, thereby shielding curcumin from potential chemical degradation. Furthermore, the use of fatty acids results in the formation of lipid digestion products. In this process, mixed micelles are responsible for the solubilization of curcumin [23]. Inspired by this inherent process, we designed curcumin-loaded SLNs based on stearic acid and mixed surfactants. Since Gelucire 48/16 is a PEG-32 (MW 1500) ester of palmitic (C16) and stearic (C18) acids, the presence of stearic acid as a solid lipid in SLNs might increase the interaction between surfactants and solid lipids and facilitate the production of SLNs. Thus, the types of surfactants were determined in terms of the saturation solubility of curcumin in surfactant solutions while the formulation study was optimized using statistical analysis.

The importance of statistical science is increasing daily due to its time and cost advantages. This experimental design involves a statistical process of analysis that helps to implement certain rules and/or detect the degree of accuracy of certain hypotheses, leading to solutions to problems. Consequently, the present study employed a CCD. Our aim with using the CCD was to analyze the evaluation of the effectiveness of factors with respect to the quality of change impact predictions. In order for parameters established during the design phase to exert a statistically significant influence on the anticipated properties, the *p*-value must be less than 0.05 [24]. Among the 17 experimental runs in this study, the particle size of the curcumin-loaded SLNs ranged from 2017 to 9996 nm. The statistical analysis revealed no direct correlation between the particle size and stirring speed. This finding aligns with the study conducted by Gupta et al. (2020), which reported that increasing the stirring speed was not highly effective in reducing the particle size below a certain threshold. While the stirring speed can influence the particle size to some extent, its impact becomes negligible beyond a certain point. In this study, the stirring speed values used were relatively close to each other (500–1000 rpm). Although a reduction in the particle size might have been observed at higher speeds, no significant effect was detected within the investigated range [25]. On the other hand, it was observed that the stirring speed was an effective variable in the experimental design because the stirring speed was identified as the most influential parameter on the zeta potential value. As the stirring speed increased, the minimum zeta potential values deemed optimal were reached more often. The EE was found to be statistically significant with respect to both the amount of curcumin and the stirring speed. As these two parameters increased, an increase in the EE was also observed. The results demonstrated that the quantity of the surfactant was a significant factor influencing the efficiency of drug loading (*p* < 0.01). The 3:1 (Brij–Gelucire) ratio, which also yielded favorable outcomes in our preliminary studies, markedly enhanced the loading efficiency. The loading capacity is influenced not only by the presence of surfactants but also by the type of lipid within the SLNs. Additionally, the lipid composition affects the zeta potential. In a study, Yeo et al. compared lauric acid, palmitic acid, and stearic acid, reporting that formulations with longer carbon chain lipids (i.e., stearic acid) produced smaller, more stable particles and a higher loading efficiency [26]. Similarly, in our study, we achieved a negatively charged zeta potential and obtained an excellent loading efficiency, likely due to the presence of stearic acid in the SLN structure. The optimum formulation showed similar characteristics to the calculated ones in terms of the particle size, zeta potential, and entrapment efficiency. Curcumin showed sustained release from SLNs without any burst effects. It reached 80.1% within six hours. This could be attributed to the slow diffusion of drug molecules from the lipid matrix to the release medium. The curcumin-loaded SLNs showed a better inhibitory effect on the cell viability. Chae et al. (2024) reported that the use of SLNs as carriers can enhance A549 cellular uptake [27]. Similarly, the developed SLNs presented a high cellular uptake with the A549 cell line.

Curcumin- and resveratrol-loaded SLNs were produced by Gumireddy et al. [19]. When different types of solid lipids (precirol, transcutol, and gelucire 50/13) were screened, the most stable formulation with the smallest particle size was achieved with the gelucire 50/13-based SLNs. However, the particle size drastically changed within 24 h. Therefore, freeze-drying was required to obtain physically stable SLNs. A curcumin release greater than 90% was also seen within 30 min [22]. It was reported that stearic acid showed an aggressive inhibitory effect on cell viability by disturbing the cellular membrane integrity [28]. Thus, using stearic acid as a solid lipid in formulations may enhance the drug efficacy. This study underlined the importance of the selection of excipients because the substances that are used to construct SLNs directly affect the final product’s characteristics. On the other hand, curcumin was released from the developed SLNs in a diffusion-controlled manner and its stability was preserved for a short period. An amount of free curcumin equal to the amount in the SLNs could not be dissolved or homogenously dispersed in PBS. With the help of the SLNs, the solubility of curcumin was increased. Free curcumin caused only a 50% cell death rate in the A549 cell line. Up to a 90% cell death rate (i.e., 10% cell viability) was reached when it was incorporated into the SLNs. Furthermore, the curcumin uptake by cells was significantly improved with the help of the SLNs.

## 4. Materials and Methods

### 4.1. Materials

Curcumin, stearic acid, Brij 35 (polyoxyethylene (23) lauryl ether), TritonX100 (polyoxyethylene octyl phenyl ether), triethanol amine (TEA), tween 80 (polyethylene glycol sorbitan monooleate), span 80 (sorbitane monooleate), benzalkonium chloride (Benz. Ch.), Sodium taurocholate (Na-Tau), and sodium lauryl sulfate (SLS) were provided from Sigma-Aldrich (Schnelldorf, Germany). β cyclodextrine sulfobutyl ethers (βCDSE) were kindly provided by Captisol. Gelucire 48/16 (polyoxyl-32 stearate) was kindly provided by Gattefossé (Mumbai, India). Propylene glycol (PG), polyethylene glycol 400, polyethylene glycol 4000, octyldodecanol (OD), poloxamer 188 (poly(ethylene glycol)-block-poly(propylene glycol)-block-poly(ethylene glycol)), macrogol cetostrearyl ether 12 (MCE12), and macrogol setotrearil ether 20 (MCE20) were kindly gifted by BASF. A549 cell line (ATCC-CCL-185) was kindly gifted from Dr. Rengin Reis (Department of Pharmaceutical Toxicology, Faculty of Pharmacy, Acibadem Mehmet Ali Aydinlar University). All organic solvents and other chemicals were analytical-grade and obtained from Merck (Darmstadt, Germany).

### 4.2. Determination Method for Curcumin

Curcumin in SLNs was detected through the validated UV method [29]. A fixed amount of curcumin was weighed and dissolved in methanol, and a set of samples was prepared within the range of 0.12–7.74 µg/mL. Spectrophotometric quantification was performed by wavelength scanning between 190 and 600 nm with a 1 nm bandwidth through UV–visible spectroscopy (Evolution 201 Spectrophotometer, Thermo Scientific, Waltham, MA, USA). Maximum absorbance values of standard samples were obtained at 422 nm. A standard calibration curve was plotted using known concentrations of curcumin. The linearity of calibration curve (r^2^) was 0.9998.

### 4.3. Saturation Solubility Study

The solubility of curcumin was studied in distilled water, cosolvents (PG and OD for the aqueous and lipid phase, respectively), and different surfactants (PEG400, PEG4000, Span 80, Poloxamer 188, βCDSE, TEA, Na-Tau, MCE12, MCE20, Tween 80, Benz. Ch., SLS, Tween80, Gelucire, TritonX100, and Brij). The aim of the present study was to investigate suitable solubilizer(s) with the ability to significantly enhance saturation solubility. Following the preparation of aqueous solutions of surfactants (0.1%) by dissolving substances in distilled water, 10 mL solutions were placed in vials. An excess amount of curcumin (5–10 mg) was added to each vial to obtain the saturated solution. Vials containing a mixture were tightly closed and stirred (1000 rpm, magnetic stirrer) for 24 h at room temperature. Samples were withdrawn and filtered through a 0.45 μm membrane filter prior to analysis. The dispersion was diluted with methanol, and we assessed the amount of curcumin using a UV–vis spectrophotometer (T70, Pharma Test, Hainburg, Germany) at 422 nm [30,31].

### 4.4. Determination of Parameters: Preliminary Study

The primary objective of the present work was to optimize the formulation. To obtain the optimum formulation through the design of experiments (DoE) approach, the relevant factors should be determined. Hence, preliminary studies were carried out to minimize the number of critical quality attributes and critical process parameters for the evaporation and purification steps. SLNs were prepared using 100, 200, or 400 mg of stearic acid. The effects of curcumin amount (10 mg or 5 mg) and ratio of surfactants (2:2 or 3:1) were also investigated. Meanwhile, the stirring rate was kept constant at 500 rpm.

### 4.5. Preparation of SLNs

Solid lipid nanoparticles (SLNs) were prepared by the solvent evaporation method [32]. In brief, stearic acid and curcumin were dissolved in ethanol and ethyl acetate, respectively. The total organic phase was fixed at 2 mL. Surfactants were dissolved in 10 mL of distilled water (aqueous phase), and the aqueous phase at 40 °C was placed under a magnetic stirrer. The organic phase was added onto the aqueous phase drop by drop. To evaporate the organic phase, the mixture was kept at 70 °C overnight. As a last step, samples were kept in the refrigerator overnight to complete the formation of SLNs. Separation of free curcumin from drug-loaded SLNs was achieved using ultracentrifugation at 60,000 rpm for 30 min. The supernatants were subjected to determine the amount of curcumin, whereas the precipitates were redispersed with distilled water and sonicated for 30 min. The obtained SLNs were kept at 4 °C for further analysis.

### 4.6. Design of Experiments

Based on the physicochemical characteristics of the curcumin nanoparticles, we defined a quality target product profile. Due to the fact that its keto–enol tautomerism depends on pH, curcumin is hydrophilic but chemically unstable under alkaline conditions, while it is stable but lipophilic (water insoluble) under acidic or neutral conditions [33]. According to the initial characterization of nanoparticle properties, we selected the surfactant type/ratio and the amount of curcumin used as critical formulation variables, whereas stirring time and rate were selected as critical process variables. A central composite design (CCD) was performed to understand the effects of all these risks on the formulation properties of the particle size, zeta potential, entrapment efficacy, and drug loading capacity. In order to reach the targeted optimized formulation, we investigated the area where the particle size and the polydispersity index were minimized and zeta potential were numerically maximized. Experimental designs were conducted using Modde (Version 10.1.1) for experimental design planning and data analysis.

The amount of active material (X_1_), surfactant concentration (X_2_), and effect of stirring in the formulation step (X_3_) were chosen as the independent variables (Table 5), which were then analyzed at three different levels for low, medium, and high levels (−1, 0, and +1, respectively) according to previous experimental results. Once the experimental responses were measured, the particle size distribution (Y_1_), zeta potential (Y_2_), and entrapment efficiency (Y_3_) were selected as the dependent variables. The objective of our study was to identify the optimal formulation, i.e., with minimal particle size (Y_1_) and zeta potential values and maximum entrapment efficiency (Y_3_). According to the experimental responses, the zeta potential values were negative, so this was minimized (and, consequently, numerically maximized).

To improve the manufactured curcumin nanoparticles, a fractional factorial design of 17 experiments was carried out (Table 1). The design included a total of 17 experimental runs: 8 factorial points (Exp. no 1–8), where all factors were at their high and low levels; 6 axial points (Exp. no 9–14), where one factor was at the midpoint while others were at high and low levels; and 3 center points (Exp. no 14–17), where all factors were at their midpoint (0) (Table 5). The center points were used to estimate error and assess model curvature.

Equation (1) shows the design fitted with a full quadratic model:

Y = bβ0 + b1A + b2B + b3C + b12AB + b13AC + b23BC + b11A2 + b22B2 + b33C2 …(1)

where Y is the predicted response(s) variable; b0 is a constant; b1, b2, and b3 were linear coefficients; b12, b13, and bβ23 were interaction coefficients between three factors; and b11, b22, and b33 were squared quadratic coefficients. It was previously stated that 2-dimensional (2D) contour plots and 3-dimensional (3D) response surface plots can be used to describe the relationship between variables and their interaction effects where all other factors are kept constant. By keeping one factor at the center level, the 3D response surface plots for particle size (Y1), PDI (Y2), and entrapment efficiency (Y3) can be plotted according to the regression model [34].

The regression coefficients calculated from the CCD are given in the equations above. For the models fitted, ANOVA tests were applied to calculate the F-values and significant probabilities of the models. The ANOVA tests confirmed the adequacy of our models, with no significant lack of fit (*p* < 0.05) and with a satisfactory coefficient of correlation (r). Based on these specified objectives, the software (Modde, Version 10.1.1, Simca) calculates the optimal formulation composition with the highest desired value. The predicted and actual responses were compared, and the percentage prediction error was computed in order to confirm that the design was applicable for optimization.

### 4.7. Particle Size and Zeta Potential Measurements

The mean particle diameter (PS) and polydispersity index (PI) values of each sample were obtained through the dynamic light scattering method using the Litesizer 500 particle analyzer (Anton Paar, Graz, Austria). Prior to analysis, distilled water was used to dilute each sample 200 times. There was no filtration stage in the analysis, which was performed at room temperature (25 °C) and at a 90° angle. Laser Doppler Velocimetry was used to measure the surface charge of SLNs. The outcomes are presented as mean ± standard deviation [35].

### 4.8. Encapsulation Efficiency

The encapsulation efficiency (EE%) for curcumin-loaded SLNs was determined by calculating the free curcumin content in the samples. Centrifugation at 60,000 rpm for 30 min was used to separate the drug that had not been encapsulated. The absorbance of the supernatant containing the unencapsulated curcumin was measured using a UV–vis spectrophotometer, as previously mentioned. The EE% was calculated by using the following equation [36]:

$$EE\%=\frac{{Drug}_{total}-{Drug}_{free}}{{Drug}_{total}}$$

### 4.9. Morphological Analysis

The nanostructures of the samples were examined morphologically with a Thermo Scientific TM Talos L120C transmission electron microscope (Thermo Fisher Scientific, Waltham, MA, USA). A copper grid with carbon coating for TEM was covered with the sample, which was then allowed to air-dry to obtain thin films. After inserting the grid in the TEM, images were captured at the appropriate magnifications [37].

### 4.10. In Vitro Release Study

The drug release of curcumin was investigated using a cellulose acetate membrane (molecular weight cutoff 12–14 kDa, Sigma-Aldrich, St. Louis, MO, USA) based on the dialysis bag method. An aliquot of 1 mL curcumin solution in ethanol or curcumin-loaded SLNs was inserted into the sealed dialysis bags and then immersed in 100 mL of preheated phosphate-buffer saline (pH 7.4) containing 20% ethanol under sink conditions [38]. The release was conducted in a multiple magnetic stirrer set at 100 rpm and 37 ± 0.5 °C. At predetermined time points, 1 mL of the sample was withdrawn and replaced with the same amount of fresh release medium. The absorbance of curcumin was determined by the UV–vis method, as described above. The amount of curcumin released from the samples was calculated using the calibration curve. For evaluation of release kinetics, the obtained release data were fitted into zeroth-order, first-order, Higuchi, and Korsmeyer–Peppas equations. The higher correlation coefficient represents the best fitted model.

### 4.11. Short-Term Stability

The produced SLNs were stored in a refrigerator. The particle size and polydispersity index for the curcumin-loaded SLNs were measured for 3 days.

### 4.12. In Vitro Cell Viability Assay

An MTT cell viability assay was conducted to test the effect of free curcumin and curcumin-loaded SLNs on cell viability. The amount of curcumin in SLNs could not be dissolved or homogenously dispersed as free curcumin in PBS due to its limited water solubility. Therefore, DMSO was used to dissolve free curcumin in order to obtain equal amounts of free curcumin with SLNs. Purified SLNs were dispersed in DMEM. Human adenocarcinoma alveolar basal epithelial cells (A549 cell line) were cultured in DMEM with 10% FBS and 1% penicillin–streptomycin, then seeded in a 96-well plate (p.31, 5 × 10^3^ cells/well). Free curcumin and SLNs loaded with curcumin were prepared in concentrations varying from 0.5 to 16 μg/mL and applied to cells in triplicates. After 24 h of incubation (37 °C, 5% CO_2_), cell viability was assessed by MTT assay by applying thiazolyl blue tetrazolium bromide solution (150 µL of 10% (*v*/*v*) 5 mg/mL) in DMEM without phenol red to each well. After 4 h of incubation (37 °C, 5% CO_2_), formazan precipitates were dissolved in 200 µL DMSO. Absorbance values at 570 and 630 nm were measured using a Multimode Plate Reader (Victor Nivo 5T, Perkin Elmer, Waltham, MA, USA). The absorbance values at 630 nm and the average absorbance value of the blank samples (DMEM) were subtracted from the 570 nm values. Cell viability was presented as percent viability relative to the negative control. Data were presented as average ± standard deviation.

### 4.13. Confocal Laser Scanning Microscopy (CLSM)

Curcumin-loaded SLNs were applied to A549 cells cultured in 24-well plates (p.31, 2 × 10^4^ cells/well). Confocal microscopy (CLSM; Zeiss LSM 900, Oberkochen, Germany) was employed to determine localization of the released curcumin within the cellular monolayer. Due to its autofluorescence properties (λ_ex_: 405 nm, λ_em_: 519 nm), curcumin was imaged without the need for additional fluorescent labeling.

## 5. Conclusions

In conclusion, the present study evaluated various solubility enhancement techniques, including the use of cosolvents, surfactants, cyclodextrin complexation, and the micellization of bile salts, to increase curcumin solubility, and our findings revealed that the most convenient method was to use a surfactant. Furthermore, we attempted to integrate this solubility enhancement technique into nanotechnology. Since curcumin is a highly lipophilic drug, we believed that the entrapment efficiency of curcumin was bound to be higher in SLNs compared to that in micelles or niosomes, which mainly consist of surfactants. Curcumin-loaded SLNs were produced successfully with stearic lipid as the solid lipid by using the solvent evaporation technique. The formulations were optimized using DoE with basic equipment and conditions, and the optimum formulation of SLNs was spherical, resulting in homogeneous nanoparticles with a high entrapment efficiency. The in vitro release study showed delayed or extended release from the curcumin-loaded SLNs compared with that with the curcumin solution. The cellular internalization and consequently the anticancer efficacy against adenocarcinomic human alveolar basal epithelial cells were improved. Therefore, the produced SLNs have very promising characteristics and can therefore be considered a new formulation of lipid-based colloidal drug delivery systems. The simplicity of production and the few production steps needed could facilitate easier standardization and upscaling, while further in vitro and in vivo investigations are desirable to evaluate the formulation’s bioavailability.

## Figures and Tables

**Table 1:** Composition of the 17 runs generated by DoE study and their obtained responses.

Run	Critical Factors			Response Variables			
	X_1_	X_2_	X_3_	PS (nm)	ZP (mV)	EE%	DL%
1	−1	−1	−1	287.9 ± 9.8	−17.3 ± 1	29.66 ± 1.92	0.15 ± 0.01
2	−1	1	−1	201.7 ± 13.5	−15.9 ± 0.9	10.73 ± 2.06	0.05 ± 0.01
3	1	−1	−1	236.9 ± 8.8	−18.3 ± 0.4	19.38 ± 1.53	0.29 ± 0.02
4	1	1	−1	398.0 ± 27.4	−10.7 ± 0.9	13.28 ± 1.58	0.20 ± 0.02
5	−1	−1	1	424.9 ± 14.8	−36.1 ± 2.1	10.07 ± 2.07	0.05 ± 0.01
6	−1	1	1	340.4 ± 13.8	−16.7 ± 0.8	28.02 ± 1.93	0.14 ± 0.01
7	1	−1	1	656.2 ± 21.0	−39.0 ± 2.6	23.02 ± 1.05	0.34 ± 0.02
8	1	1	1	512.3 ± 33.4	−31.8 ± 1.4	23.95 ± 1.47	0.36 ± 0.02
9	0	−1	0	929.5 ± 50.9	−31.5 ± 2.4	24.85 ± 1.26	0.25 ± 0.01
10	0	1	0	999.6 ± 51.2	−30.2 ± 1.1	21.74 ± 1.29	0.22 ± 0.01
11	−1	0	0	780.0 ± 33.2	−30.6 ± 1.1	24.90 ± 0.60	0.12 ± 0.01
12	1	0	0	509.2 ± 27.3	−21.9 ± 1.6	28.36 ± 1.01	0.42 ± 0.02
13	0	0	−1	364.8 ± 9.7	−24.1 ± 1.1	28.11 ± 1.24	0.28 ± 0.01
14	0	0	1	507.2 ± 20.7	−22.5 ± 1.1	28.76 ± 1.23	0.29 ± 0.01
15	0	0	0	354.1 ± 44.5	−17.8 ± 4.1	34.31 ± 1.19	0.34 ± 0.01
16	0	0	0	356.3 ± 10.8	−23.7 ± 1.0	33.33 ± 1.20	0.33 ± 0.01
17	0	0	0	339.5 ± 7.4	−25.2 ± 1.4	20.60 ± 1.30	0.21 ± 0.01

X_1_: curcumin amount; X_2_: surfactant ratio; X_3_: stirring speed.

**Table 2:** Models’ coefficients for different responses.

		Coefficients			
		ZP (mV)	PS (nm)	EE%	DL%
bo	Constant	−25.097 **	603.585 **	29.361 **	0.295 **
b1	Curcumin (mg)	3.690	−8.340	−0.926	−0.011
b2	Surfactant (%)	−0.510	27.770	0.461	0.110 **
b3	Stirring (rpm)	−5.980 *	95.170	1.266	0.021
b1 × b1	Cur × Cur	−3.605	220.752	−6.027	−0.061
b2 × b2	Sur × Sur	0.995	−99.198	−2.692	−0.026
b3 × b3	Stir × Stir	3.945	−307.798	−0.887	−0.011
b1 × b2	Cur × Sur	−0.750	23.488	−0.524	−0.008
b1 × b3	Cur × Stir	2.200	−37.913	5.489 *	0.038
b2 × b3	Sur × Stir	−2.775	32.238	2.076	0.028

* *p* values < 0.05 and ** *p* < 0.01.

**Table 3:** Measured and calculated results from DoE study for the optimum formulation.

	PS (nm)	ZP (mV)	EE (%)	DL (%)
**Optimum formulation**	389.31 ± 9.95	−27.50 ± 0.80	28.95 ± 4.36	0.27 ± 0.03
**Calculated**	369.66	−30.85	28.28	0.41

**Table 4:** Release kinetic equations.

	Drug	r^2^	e	Intercept
**0th-order kinetics**	Free	0.658	3	20.723
	Loaded	0.915		7.080
**1st-order kinetics**	Free	0.325	88	2.243
	Loaded	0.430		1.592
**Higuchi**	Free	0.799	70	2.534
	Loaded	0.962		−8.108
**Korsmeyer–Peppas**	Free	0.842	87	2.089
	Loaded	0.743		1.248

**Table 5:** Factors and their levels in DoE for curcumin-loaded SLNs.

Independent Factors	Level Used		
	−1	0	+1
X_1_: The amount of curcumin (mg)	2.5	5	7.5
X_2_: The ratio of surfactants (Brij–Gelucire)	400:0	200:200	300:100
X_3_: The stirring speed (rpm)	500	700	1000

## Data Availability

The data are contained within the article.

## Abbreviations

The following abbreviations are used in this manuscript:

SLN	Solid lipid nanoparticle
TEA	Triethanol amine
Benz. Ch.	Benzalkonium chloride
Na-Tau	Sodium taurocholate
SLS	Sodium lauryl sulfate
βCDSE	β cyclodextrine sulfobutyl ethers
PG	Propylene glycol
OD	Octyldodecanol
MCE12	Macrogol cetostrearyl ether 12
MCE20	Macrogol setotrearil ether 20
DoE	Design of experiment
CCD	Central composite design
2D	2-dimensional
3D	3-dimensional
PS	Particle diameter
PI	Polydispersity index
EE	Encapsulation efficiency
CLSM	Confocal Laser Scanning Microscopy
HLB	Hydrophilic–lipophilic balance
